# Supplementary material for: MetaRibo-Seq measures translation in microbiomes
Source: Nat Commun. 2020 Jun 29;11:3268. doi: 10.1038/s41467-020-17081-z (PMC7324362; doi:10.1038/s41467-020-17081-z)
Supplement: Supplementary file 10 — Supplementary Data 7 [file 41467_2020_17081_MOESM10_ESM.zip › File2/Confidence_High_Taxonomy/209483_out.krona.html]

Javascript must be enabled to view this page.

members
magnitude
magnitudeUnassigned
count
unassigned
taxon
rank

209483\_out

207

207
superkingdom
2

205
phylum
976

200643
class
205

order
171549
205

family
815
200


SRS078419\_contig\_number\_17868SRS893292\_contig\_number\_381
2
200
816
genus


SRS077086\_contig\_number\_128
1
species
2292928


SRS143598\_contig\_number\_456
1946092
species
1

6
species
818

SRS016753\_contig\_number\_121SRS016989\_contig\_number\_376SRS043001\_contig\_number\_597SRS045739\_contig\_number\_125SRS075021\_contig\_number\_285SRS075078\_contig\_number\_9945


SRS103987\_contig\_number\_3139
species
674529
1

1
457387
species

SRS013687\_contig\_number\_3391

species
1946089
25

SRS013951\_contig\_number\_20140SRS014948\_contig\_number\_23188SRS015190\_contig\_number\_5000SRS016018\_contig\_number\_655SRS016495\_contig\_number\_2024SRS024132\_contig\_number\_7761SRS045004\_contig\_number\_1701SRS058770\_contig\_number\_127SRS062701\_contig\_number\_3909SRS075878\_contig\_number\_2135SRS076929\_contig\_number\_5009SRS077641\_contig\_number\_contig-100\_2309.117964SRS1041137\_contig\_number\_28247SRS104636\_contig\_number\_14691SRS1055083\_contig\_number\_8200SRS140513\_contig\_number\_1600SRS143876\_contig\_number\_2254SRS144603\_contig\_number\_14944SRS147088\_contig\_number\_contig-100\_1682.53508SRS147139\_contig\_number\_221SRS147977\_contig\_number\_2643SRS149181\_contig\_number\_13823SRS150029\_contig\_number\_17149SRS893187\_contig\_number\_16468SRS893342\_contig\_number\_8053


SRS1041142\_contig\_number\_452
1
species
2292944


SRS016095\_contig\_number\_6920SRS1055038\_contig\_number\_164
2
291645
species

9
46506
species

SRS015579\_contig\_number\_437SRS023971\_contig\_number\_393SRS048164\_contig\_number\_12630SRS049995\_contig\_number\_12466SRS055017\_contig\_number\_7463SRS057478\_contig\_number\_987SRS077454\_contig\_number\_1897SRS104311\_contig\_number\_7446SRS142599\_contig\_number\_83


SRS046369\_contig\_number\_1079SRS144297\_contig\_number\_10672
2
species
28116

1
2292946
species

SRS021219\_contig\_number\_1683

2
821
species

SRS014313\_contig\_number\_4093SRS049402\_contig\_number\_6155


SRS011084\_contig\_number\_10334SRS011239\_contig\_number\_48SRS011302\_contig\_number\_3899SRS012273\_contig\_number\_30330SRS013098\_contig\_number\_18235SRS013158\_contig\_number\_960SRS013940\_contig\_number\_44SRS013951\_contig\_number\_338SRS014313\_contig\_number\_114SRS014412\_contig\_number\_14246SRS014534\_contig\_number\_52SRS014613\_contig\_number\_10071SRS014979\_contig\_number\_30866SRS015264\_contig\_number\_12813SRS015578\_contig\_number\_9060SRS015663\_contig\_number\_29316SRS015890\_contig\_number\_1800SRS016495\_contig\_number\_15141SRS016840\_contig\_number\_34SRS016897\_contig\_number\_230SRS017521\_contig\_number\_6530SRS017745\_contig\_number\_2478SRS017821\_contig\_number\_963SRS018541\_contig\_number\_4776SRS018623\_contig\_number\_1730SRS018984\_contig\_number\_4224SRS019286\_contig\_number\_2330SRS019445\_contig\_number\_19464SRS019693\_contig\_number\_3687SRS020233\_contig\_number\_3429SRS020271\_contig\_number\_1374SRS020508\_contig\_number\_74SRS021484\_contig\_number\_3637SRS022524\_contig\_number\_8921SRS022713\_contig\_number\_1474SRS023914\_contig\_number\_13098SRS024331\_contig\_number\_1544SRS042284\_contig\_number\_2273SRS042628\_contig\_number\_30294SRS042966\_contig\_number\_8429SRS043411\_contig\_number\_10815SRS045645\_contig\_number\_3959SRS046068\_contig\_number\_292SRS046502\_contig\_number\_11695SRS047433\_contig\_number\_15331SRS048981\_contig\_number\_906SRS049896\_contig\_number\_2703SRS049900\_contig\_number\_14677SRS050299\_contig\_number\_315SRS050422\_contig\_number\_480SRS050520\_contig\_number\_145SRS050752\_contig\_number\_12869SRS050925\_contig\_number\_2420SRS050998\_contig\_number\_5317SRS051031\_contig\_number\_5550SRS051610\_contig\_number\_10754SRS052576\_contig\_number\_contig-100\_787.788SRS053214\_contig\_number\_11935SRS056273\_contig\_number\_1528SRS056537\_contig\_number\_56231SRS063370\_contig\_number\_414SRS063489\_contig\_number\_1021SRS063928\_contig\_number\_290SRS064645\_contig\_number\_207SRS065397\_contig\_number\_1100SRS065504\_contig\_number\_10764SRS074586\_contig\_number\_4397SRS075716\_contig\_number\_278SRS075821\_contig\_number\_5303SRS076756\_contig\_number\_1175SRS076804\_contig\_number\_26SRS076876\_contig\_number\_117SRS076976\_contig\_number\_4034SRS076976\_contig\_number\_4934SRS077024\_contig\_number\_3115SRS077194\_contig\_number\_9037SRS077231\_contig\_number\_3018SRS077294\_contig\_number\_430SRS077589\_contig\_number\_276SRS077730\_contig\_number\_2167SRS078176\_contig\_number\_12954SRS097889\_contig\_number\_314SRS097958\_contig\_number\_11890SRS098061\_contig\_number\_1802SRS098571\_contig\_number\_15137SRS098655\_contig\_number\_160SRS100021\_contig\_number\_10184SRS101268\_contig\_number\_36SRS1041033\_contig\_number\_25410SRS1041036\_contig\_number\_53SRS1041037\_contig\_number\_1889SRS1041039\_contig\_number\_21189SRS1041095\_contig\_number\_242SRS1041112\_contig\_number\_332SRS1041116\_contig\_number\_3695SRS1041130\_contig\_number\_1022SRS1041133\_contig\_number\_20141SRS1041134\_contig\_number\_228SRS1041144\_contig\_number\_3028SRS1041147\_contig\_number\_435SRS1041157\_contig\_number\_5475SRS1041164\_contig\_number\_758SRS104400\_contig\_number\_9379SRS104975\_contig\_number\_15472SRS1055022\_contig\_number\_514SRS1055095\_contig\_number\_86SRS142781\_contig\_number\_1013SRS143085\_contig\_number\_1045SRS143372\_contig\_number\_1761SRS143598\_contig\_number\_353SRS143598\_contig\_number\_1741SRS143722\_contig\_number\_2079SRS143895\_contig\_number\_contig-100\_265.263672SRS144183\_contig\_number\_3112SRS144297\_contig\_number\_6783SRS144537\_contig\_number\_28502SRS145308\_contig\_number\_646SRS146764\_contig\_number\_4964SRS146764\_contig\_number\_5095SRS146812\_contig\_number\_25978SRS147271\_contig\_number\_30509SRS147377\_contig\_number\_1874SRS147614\_contig\_number\_3245SRS148091\_contig\_number\_144SRS148319\_contig\_number\_171SRS148970\_contig\_number\_619SRS149075\_contig\_number\_817SRS893230\_contig\_number\_3476SRS893252\_contig\_number\_135SRS893253\_contig\_number\_46SRS893256\_contig\_number\_404SRS893270\_contig\_number\_7078SRS893300\_contig\_number\_8114SRS893380\_contig\_number\_363SRS893382\_contig\_number\_1599SRS971275\_contig\_number\_3343SRS971276\_contig\_number\_3345
species
820
137

246787
species
5

SRS016132\_contig\_number\_790SRS018541\_contig\_number\_730SRS019685\_contig\_number\_4289SRS143780\_contig\_number\_160SRS893172\_contig\_number\_576

species
626931
1

SRS1041145\_contig\_number\_10164

371601
species
1

SRS052078\_contig\_number\_290

47678
species
1

SRS148196\_contig\_number\_19267


SRS101376\_contig\_number\_11164
329854
species
1

1
family
1853231

1
genus
283168

1
species
626933

SRS013476\_contig\_number\_39423

family
2005525
4

4
genus
375288


SRS144362\_contig\_number\_968
1
species
2293116

1
species
1947048

SRS043768\_contig\_number\_14948


SRS014235\_contig\_number\_30348SRS893295\_contig\_number\_99
2
46503
species

2
phylum
1239

2
class
186801

186802
order
2
1

SRS893373\_contig\_number\_12319

1
family
541000

1263
genus
1


SRS019910\_contig\_number\_7729
species
2292375
1
